# A Biomechanical Analysis of Wide, Medium, and Narrow Grip Width Effects on Kinematics, Horizontal Kinetics, and Muscle Activity on the Sticking Region in Recreationally Trained Males During 1-RM Bench Pressing

**DOI:** 10.3389/fspor.2020.637066

**Published:** 2021-01-22

**Authors:** Stian Larsen, Olav Gomo, Roland van den Tillaar

**Affiliations:** Department of Sports Science and Physical Education, Nord University, Levanger, Norway

**Keywords:** strength, force, electromyography, kinetics, kinematics

## Abstract

Grip width has been found to affect lifting performance, especially around the sticking region; however, little is known about the kinetics and muscle activities that could explain these differences in performance. This study aimed to investigate the effects of grip width on the joint, barbell kinematics, and horizontal kinetics, analyzed in tandem with the effects of muscle activation around the sticking region in the one repetition maximum (1-RM) barbell bench press. Fourteen healthy bench press-trained males (body mass: 87.8 ± 18.4, age: 25 ± 5.4) performed 1-RM with a small, medium, and wide grip width. The participants bench pressed 109.8 ± 24.5 kg, 108.9 ± 26.4 kg, and 103.7 ± 24 kg with the wide, medium, and narrow grip widths. Furthermore, the wide grip width produced 13.1–15.7% lateral forces, while the medium and narrow grip widths produced 0.4–1.8 and 8.5–10.1% medially directed forces of the vertical force produced during the sticking region, respectively. Horizontal forces did not increase during the sticking region, and the resultant forces decreased during the sticking region for all grip widths. The wide and medium grip widths produced greater horizontal shoulder moments than the narrow grip width during the sticking region. Hence, the wide and medium grip widths produced similar shoulder and elbow joint moments and moment arm at the first located lowest barbell velocity. Furthermore, triceps medialis muscle activity was greater for the medium and narrow grip widths than the wide grip width. This study suggests that the sticking region for the wide and medium grip widths may be specific to the horizontal elbow and shoulder joint moments created during this region. Therefore, when the goal is to lift as much as possible during 1-RM bench press attempts among recreationally trained males, our findings suggest that bench pressing with a wide or medium grip width may be beneficial.

## Introduction

**Figure 1:**
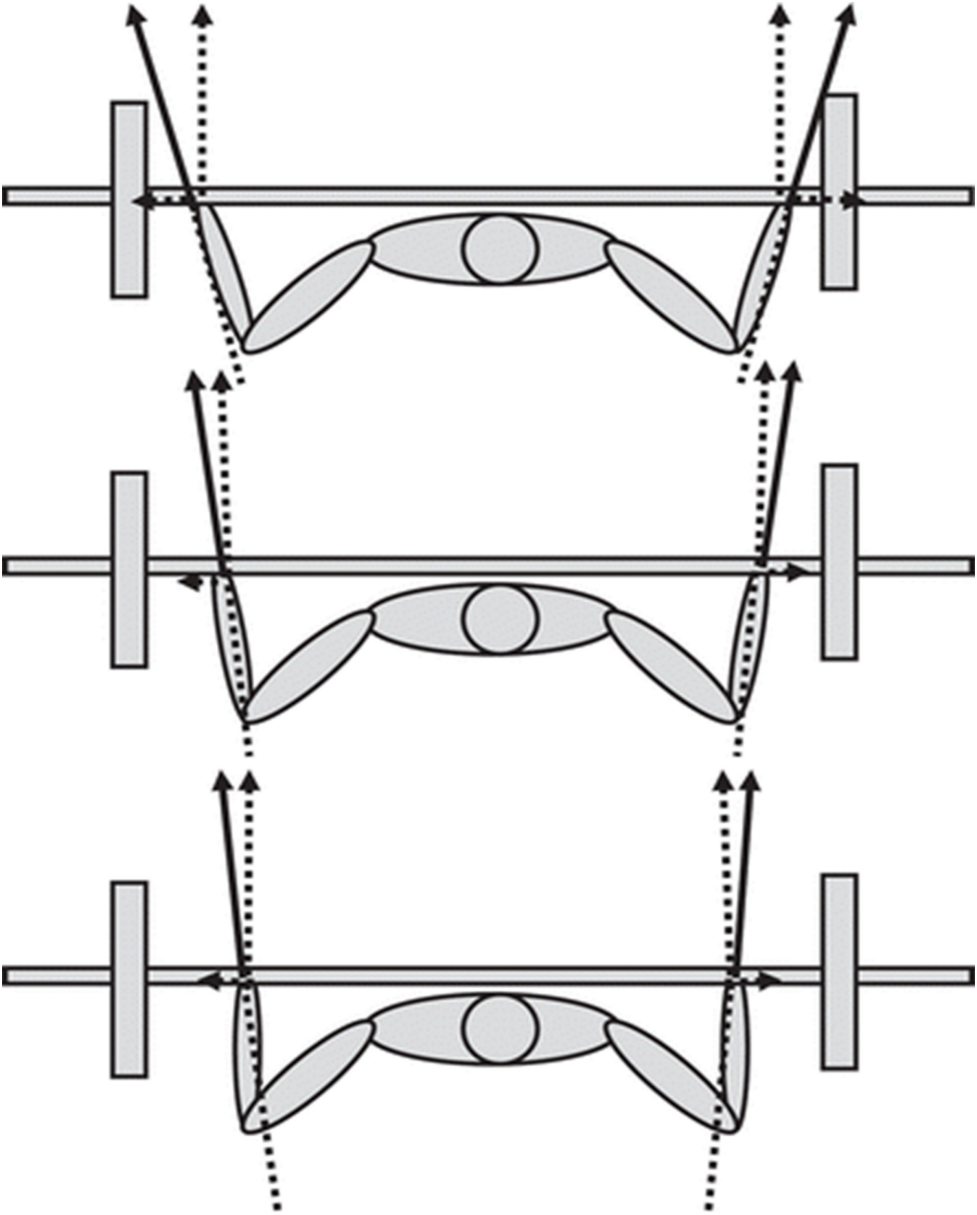
Schematic overview of lateral and vertical force production with same total force production but with different grip widths. Adapted from Gomo and van den Tillaar (2016).

The barbell bench press is a popular exercise for the upper limbs when one's goal is to enhance strength. For powerlifters, the bench press is the main exercise for measuring maximal upper-body strength during a competition. To measure the strongest athlete in a powerlifting competition, the one repetition maximum (1-RM) is evaluated in the squat, bench press, and deadlift and the highest successful lift in each exercise is added together to arrive at the total amount of kilograms lifted (Wilk et al., 2020). However, when a barbell load is over 90% of the 1-RM in single lifts, a sticking region is reported to occur (Newton et al., 1997; Duffey and Challis, 2011). The sticking region is referred to as the region in which most lifts fail during training and competition, and is defined as the region from the initial peak upwards velocity to the first local minimum velocity of the barbell also called the sticking point (Madsen and McLaughlin, 1984; Elliott et al., 1989; van den Tillaar and Ettema, 2010). Lander et al. (1985) postulated that failure will most likely occur in this region because the lifters' ability to generate force is lower than the magnitude of the barbell load. Therefore, several studies have investigated the underlying mechanisms behind the sticking region to enhance our understanding regarding what causes the sticking region, as well as how to surpass the region to complete the lift (van den Tillaar and Ettema, 2009, 2010; van den Tillaar et al., 2012; van den Tillaar and Saeterbakken, 2013; van den Tillaar, 2015, 2019; Gomo and van den Tillaar, 2016; Kompf and Arandjelović, 2016; Saeterbakken et al., 2020). Elliott et al. (1989) and Madsen and McLaughlin (1984) proposed that the sticking region occurs because the muscles involved are in a poor mechanical force position, which reduces their capability to exert force in this region. van den Tillaar et al. (2012) tested this hypothesis by conducting the 1-RM bench press and isometric bench press at twelve different heights from the sternum and found a decrease in force output in the sticking region for both conditions. The authors suggested that the occurrence of the sticking region could be due to the force-length relationship of the involved muscles, which created a poor mechanical force position in the sticking region. To test this hypothesis, Gomo and van den Tillaar (2016) performed a study using three different grip widths, surmising that if there was a biomechanically poor region for force production, it would occur at the same joint angle in all three lifts, since the joint angle represents the length of the muscles and would approximately be of equal length to the muscles involved. The main finding from Gomo and van den Tillaar (2016), however, was that the sticking region was not angle-specific. Nevertheless, their hypothesis cannot be rejected yet because only vertical forces were measured, even though the total force output is a combination of both vertical and horizontal forces. Duffey and Challis (2011) found that there could be a large horizontal force component in the bench press that peaks around 26.3% during maximal lifts in the ascend phase among novice lifters. This means that the total force vector could influence the moments around the elbow from flexion to an extension moment. Duffey and Challis (2011) therefore suggested that the horizontal forces during bench press result from the muscles' engagement in order to generate vertical force; they did not, however, investigate the effect of a horizontal force component in the sticking region. Therefore, the investigation of such an effect utilizing different grip widths of a 1-RM barbell bench press could provide further information regarding whether or not the sticking region is influenced by horizontal forces to a significant degree, which may affect one's ability to generate vertical forces (Figure 1).

Since a poor biomechanical region would affect the muscles' capability to exert force, electromyographic muscle activity (EMG) has been examined in regards to the sticking region in several studies (Elliott et al., 1989; van den Tillaar and Ettema, 2009, 2010; van den Tillaar et al., 2012), but the effect of grip width on muscle activity in the sticking region has not been adequately investigated. Considering EMG activity during different grip widths along with the horizontal forces around the sticking region could provide more detailed information about how these muscles generate forces in the sticking region during different grip widths, and how to manipulate the sticking region by choosing a grip width that supports individual strength and weaknesses.

This study aimed to investigate the effects of grip width on the joint, barbell kinematics, and horizontal kinetics, analyzed in tandem with the effects of muscle activation around the sticking region in the 1-RM barbell bench press. It was hypothesized that the horizontal force output increased in the sticking region, but that the total force would be the same. An additional hypothesis was that the horizontal force would act laterally on the wide grip bench press condition and medially on the narrow grip bench press condition, potentially affecting the muscle activity of the prime movers differently in the sticking region due to the different moments on the elbow and shoulder.

## Methods

### Experimental Design

To investigate the effect of grip width upon joint and barbell kinematics, kinetics (including vertical, horizontal, and resultant forces; angle, and magnitude; arm joint moment; and moments on the elbow and shoulder), and muscle activation around the sticking region, a within-subjects, repeated-measures design was used. The three grip widths were used as independent variables. The muscle activity during the pre-sticking, sticking, and post-sticking regions, together with the joint and barbell kinematics and forces with direction at the different events during the ascending phase of the bench press lifts, served as dependent variables.

### Participants

Fourteen healthy recreational bench press-trained males (body mass: 87.8 ± 18.4, age: 25 ± 5.2) were recruited for the study with at least 3 years of bench press training experience. Inclusion criteria were that participants did not have an injury that could reduce maximal performance, and were able to bench press 1.2 times their own bodyweight with their preferred bench press grip width. A written consent was obtained from each participant before the study. The study complied with the current ethical regulations for research, was approved by the Norwegian Center for Research Data, and was conducted in accordance with the Declaration of Helsinki.

## Procedures

The participants were randomly assigned an order to perform the three grip widths, which were defined as follows: wide as 1.7 times bi-acromial distance (0.71 ± 0.06 m), narrow as the bi-acromial distance (0.40 ± 0.04 m), and medium as 1.4 times bi-acromial distance (0.56 ± 0.04 m). After a general warm-up, which included as many repetitions as the participant wanted with just the barbell, the participants conducted a standardized warm-up protocol with the first grip width as follows: 8 reps at 40% of the self-estimated one repetition maximum (1-RM_est_), 6 reps at 60% of the 1-RM_est_, 3 reps at 70% of the 1-RM_est_, and 2 reps at 80% of the 1-RM_est_. The 1-RM_est_ was the weight that the subject himself estimated to be his one repetition maximum at that grip width. After the warm-up protocol, the subjects were tested at 95% of the 1-RM_est_ and 100% of the 1-RM_est_. If the 100% lift was successful, the weight was raised by 2.5 or 5 kg, depending on the lifters' feedback, until a missed or a near-missed lift occurred. Three attempts were performed in total with each grip width, and the highest completed lift was used for further analysis.

After the first 1-RM was established, the lifter was instructed to perform a warm-up set with 3 reps at 80% of the 1-RM_est_ at the second grip width. This set was supposed to work as an adaptation set to the new width. Then, the lifter performed the same testing routine as they did with the first grip width. The same procedure of one adaptation set at 80% of the 1-RM_est_ and then a 95% and 100% test (plus possible increases if the lift was not maximum) was conducted with the last grip width. The lifter was given 3–5 min of rest between each attempt. The subjects performed the bench press according to the rules and regulations set by the International Powerlifting Federation, except that the requirement for a full stop on the chest was removed; they were allowed to touch and press, but no bounce of the barbell was allowed. Descent movement velocity was volitional or self-selected with an average around 1 ± 0.5 m/s.

## Recordings

**Figure 2:**
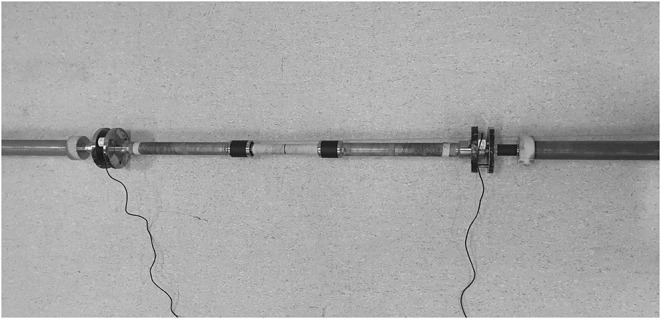
A specially made barbell that included two force cells (Ergotest Technology AS, Langesund, Norway) that could measure the horizontal forces.

Before the warm-up, electrodes with a contact diameter of 11 mm and a distance of 20 mm from center to center were placed on the dominant side on seven muscles as per the recommendations described by SENIAM (Hermens et al., 2000): The upper and lower pectoralis major, ~4 cm from the middle of the axillary fold (Schick et al., 2010); the triceps lateralis and medialis, approximately at halfway on the line between the posterior crista of the acromion, and the olecranon at 2 finger-widths lateral to the line for the lateral head and 1 finger width medial for the medial head; the deltoideus anterior, at 1.5 cm distal and anterior to the acromion; the medius, between the acromion and the lateral epicondyle of the elbow; the posterior, at about two finger breaths behind the angle of the acromion; and the biceps brachii, on the line between the medial acromion and the fossa cubit, which was about one-third the distance from the fossa cubit. First, the skin was shaved, washed with alcohol, and abraded before the electrodes were placed. EMG-activity was recorded with Musclelab10.5.69.4823 (Ergotest Technology AS; Langesund, Norway). Raw EMG signals were amplified and filtered with a preamplifier located near the pick-up point. Signals were passed through high pass and low pass (500, 20 Hz) filters. Furthermore, the raw EMG signals were converted to root of mean square (RMS) signals using a hardware circuit network (frequency response 450 kHz, averaging constant 12 ms; total error ± 0.5%) with a common rejection rate of 106 dB. A specially made barbell was used that included two force cells (Ergotest Technology AS, Langesund, Norway) that could measure the horizontal forces (Figure 2). A force plate (Ergotest Technology AS, Langesund Norway) was placed directly under the bench and the participant's feet were placed in line with the barbell to measure the vertical forces during the lift at a sampling rate of 1,000 Hz. EMG signals, force data, and barbell kinematics were synchronized using Musclelab and analyzed by the software v10.5.69.4823 (Ergotest Technology AS; Langesund, Norway). To investigate possible differences in EMG activity during the 1-RM bench press conditions, the average RMS was calculated for the pre-sticking, sticking, and post-sticking regions. The pre-sticking region was from the lowest barbell point (v_0_) to the first peak of barbell velocity (v_max1_). The sticking region was from the first peak of barbell velocity (v_max1_) to the first located lowest barbell velocity (v_min_). The post-sticking region was from the first located lowest barbell velocity (v_min_) to the second maximal peak of barbell velocity (v_max2_) (van den Tillaar et al., 2012).

A three-dimensional (3D) motion capture system (Qualisys, Gothenburg, Sweden) with eight cameras was used to track markers at a frequency of 500 Hz, creating a 3D positional measurement. The reflective markers were placed on the lateral tip of the acromion, the medial and lateral epicondyle of the elbows, and the styloid process of the radius and ulna, and two markers were placed in the middle of the bar 20 cm apart to track the barbell displacement. The points that were analyzed for the joint (shoulder flexion and abduction and elbow extension, Figure 1) and barbell kinematics were located at the start of the upwards movement (v_0_), the first peak barbell velocity (v_max1_), the first local minimum velocity (v_min_), and the second peak velocity (v_max2)_ because these points define the pre-sticking, sticking and post-sticking regions. Barbell position (horizontal and vertical displacement), velocity, time of occurrence of the different events, and joint angles at the shoulder and elbow joints were calculated in Visual3D v6.03.6 (C-motion, Germantown, MD, USA). Shoulder flexion/abduction and elbow extension were estimated via the angles calculated from lines formed between the centers of the reflective markers by van den Tillaar and Ettema (2009). Moment arms and moments on the elbow and shoulder at the different events in the three grip width conditions were calculated by using the different angles measured in Visual 3D and force measurements. Calculation was performed in Excel 2017 (Microsoft Corp, Redmond, WA, USA).

## Statistical Analyses

Normality was tested using the Shapiro-Wilks test of normality. To compare barbell kinematics (velocity, lifting time, and displacement), a one-way analysis of variance (ANOVA) with repeated measures on grip condition (wide, medium, and small) for each event was performed. To investigate forces (horizontal, vertical, and resultant), moment arms, joint moments, and the joint angle between the three bench press conditions, a 3 (condition: wide, medium, and narrow) ^*^ 4 (event: v_0_, v_max1_, v_min_, and v_max2_) ANOVA with repeated measurements was used. To assess differences in muscle activity, a 3 (condition: wide, medium, and narrow) ^*^ 3 (region: pre-sticking, sticking, and post-sticking) ANOVA with repeated measurements for each of the eight upper-limb muscles was applied. A *post hoc* test was conducted with a Bonferroni correction to identify where the eventual differences in kinematics, kinetic, and muscle activity occurred. If the sphericity assumption was violated, *p*-values of the Greenhouse-Geisser adjustment were reported. All results are presented as mean ± standard deviation (SD), and the alpha level was set at p < 0.05. The effect size was evaluated as η^2^ (Eta partial squared) where 0.01 < η^2^ < 0.06 constitutes a small effect, 0.06 < η^2^ < 0.14 constitutes a medium effect, and η^2^ > 0.14 constitutes a large effect (Cohen, 1988). Statistical analyses were performed in SPSS version 25.0 (SPSS, Inc., Chicago IL, USA).

## Results

### Barbell Kinematics

The participants lifted 109.8 ± 24.5, 108.9 ± 26.4, and 103.7 ± 24 kg in the wide, medium, and narrow bench press conditions. The 1-RM lift for the wide and medium grips was significantly higher than the narrow grip (*F* = 24.9, *p* < 0.001, η^2^ = 0.68). Vertical displacement (*F* ≥ 10.7, *p* ≤ 0.004, η^2^ ≥ 0.45) and maximal velocities (*F* ≥ 3.9, *p* ≤ 0.032, η^2^ ≥ 0.23) were significantly different between the three conditions, whereas no significant differences were found in horizontal displacement (*F* ≤ 1.22, *p* ≥ 0.31, η^2^ ≤ 0.09), minimal velocity (*F* = 1.6, *p* = 0.22, η^2^ = 0.11), or interval times (*F* ≤ 1.5, *p* ≥ 0.225, η^2^ ≤ 0.11) at the different events between the three conditions (Table 1). *Post hoc* compression revealed that maximal velocity was increased at v_max1_ from wide-medium-narrow conditions and only significantly higher in narrow grip condition at v_max2_ as compared with the wide condition. Vertical displacement was also increased from the wide-medium-narrow condition at v_0_. During the ascending phase, vertical displacement was higher at nearly every event when a narrow grip was used as compared with the other conditions; in the wide condition, the displacement at each event was less (Table 1).

**Table 1:** Mean (SD) vertical, horizontal barbell displacement, barbell velocity and time of occurrence of the different events in the wide, medium, and narrow grip bench press.

**Event**	**Grip condition**	**Vertical displacement from start and v_**0**_ (m)**	**Horizontal displacement from start (m)**	**Velocity (m/s)**	**Interval time (s)**
v_0_	Wide	0.338 ± 0.066^*^	0.125 ± 0.035	0	0
	Medium	0.373 ± 0.060^*^	0.131 ± 0.045	0	0
	Narrow	0.399 ± 0.064^*^	0.128 ± 0.043	0	0
v_max1_	Wide	0.033 ± 0.019	0.109 ± 0.043	0.233 ± 0.076^*^	0.227 ± 0.124
	Medium	0.038 ± 0.011	0.121 ± 0.043	0.284 ± 0.082^*^	0.233 ± 0.099
	Narrow	0.051 ± 0.014^*^	0.123 ± 0.041	0.302 ± 0.083^*^	0.231 ± 0.125
v_min_	Wide	0.154 ± 0.053^*^	0.056 ± 0.049	0.066 ± 0.064	0.961 ± 0.401
	Medium	0.186 ± 0.049^*^	0.056 ± 0.052	0.095 ± 0.058	0.972 ± 0.422
	Narrow	0.219 ± 0.056^*^	0.062 ± 0.042	0.060 ± 0.060	0.958 ± 0.466
v_max2_	Wide	0.314 ± 0.052^*^	−0.018 ± 0.043	0.240 ± 0.055^†^	1.374 ± 0.795
	Medium	0.345 ± 0.050	−0.010 ± 0.049	0.283 ± 0.109	1.223 ± 0.606
	Narrow	0.368 ± 0.076	−0.009 ± 0.040	0.361 ± 0.122^†^	1.026 ± 0.479

* *Indicates a significant difference with all other grip conditions on a p < 0.05 level*.

*Indicates a significant difference between these two grip conditions on a p < 0.05 level*.

### Joint Kinematics

**Figure 3:**
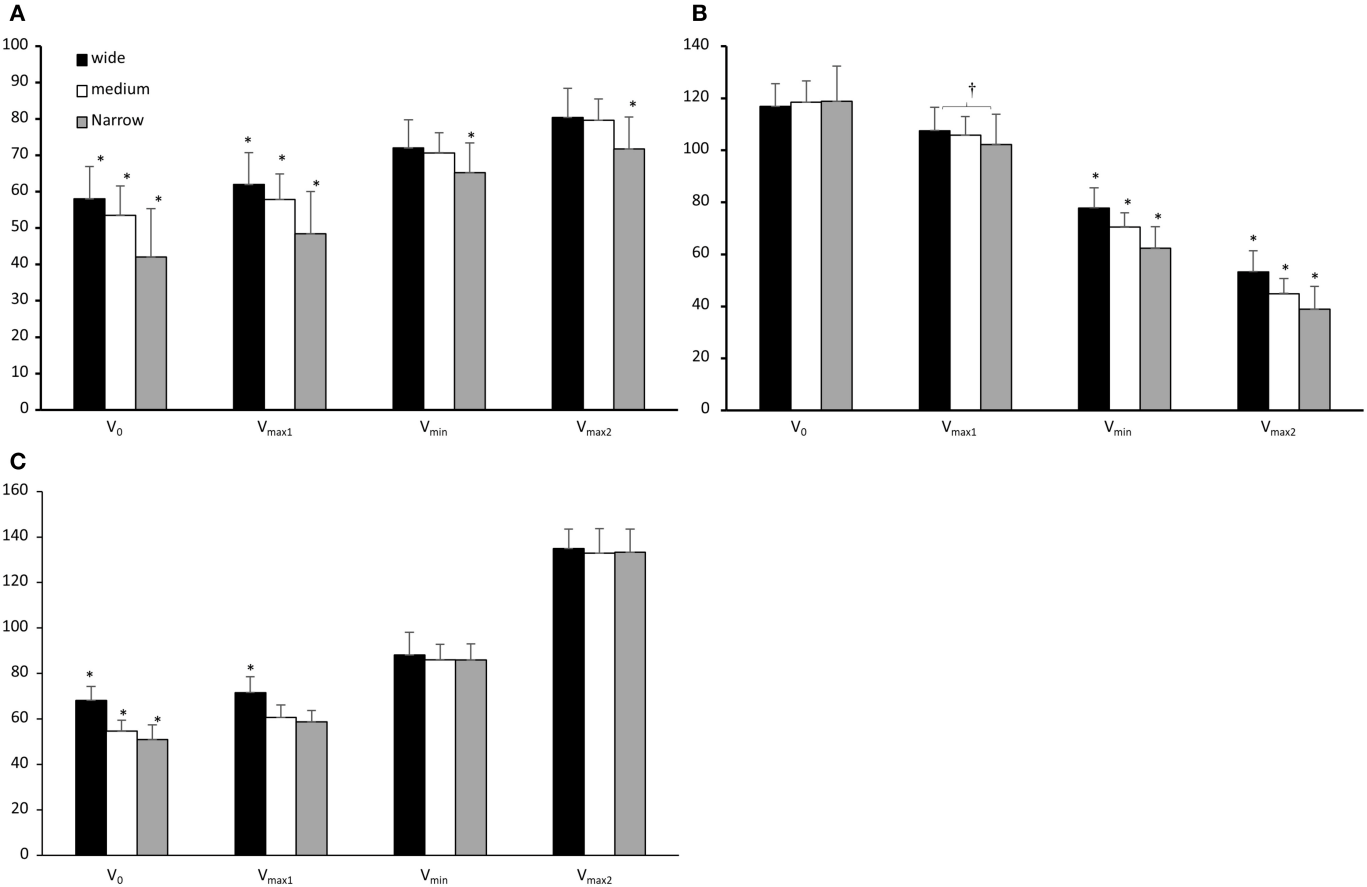
**(A)** Shoulder abduction, **(B)** shoulder flexion, and **(C)** elbow extension expressed as mean (SD) in v_0_, v_min_, v_max2_, v_0_, and v_max1_ for the wide, medium, and narrow grip bench press conditions. *Indicates a significant difference when compared with the other two grip widths. ^†^Indicates a significant difference between the two specified grip widths.

Shoulder abduction and elbow extension angles increased significantly, while shoulder flexion decreased significantly from one event to the next (*F* ≥ 211, *p* < 0.001, η^2^ ≥ 0.94). Significant differences were found for the shoulder abduction, shoulder flexion, and elbow extension angles between the three grip widths at the different events (*F* ≥ 15.54, *p* < 0.001, η^2^ ≥ 0.54) and significant interaction effects (event*grip width) for all three joint angles (*F* ≥ 3.51, *p* ≤ 0.016, η^2^ ≥ 0.21). *Post hoc* comparisons showed that the narrow grip width had a significantly lower shoulder abduction angle than the medium and wide grip widths in all events, while the medium grip width had a significantly lower shoulder abduction than the wide grip width in v_0_ and v_max1_ (Figure 3). Shoulder flexion was significantly different in the last two events between all three grip widths: the narrow grip width had the smallest shoulder flexion, followed by the medium and wide grip widths (Figure 3). Additionally, shoulder flexion at v_max1_ was significantly less between the narrow grip and the wide grip conditions. The elbow extension angle differed at v_0_ between all conditions: the wide grip had the largest extension angle, followed by the medium and narrow grips (Figure 3). At v_max1_, elbow extension was significantly larger than with the other two grips.

### Kinetics

**Figure 4:**
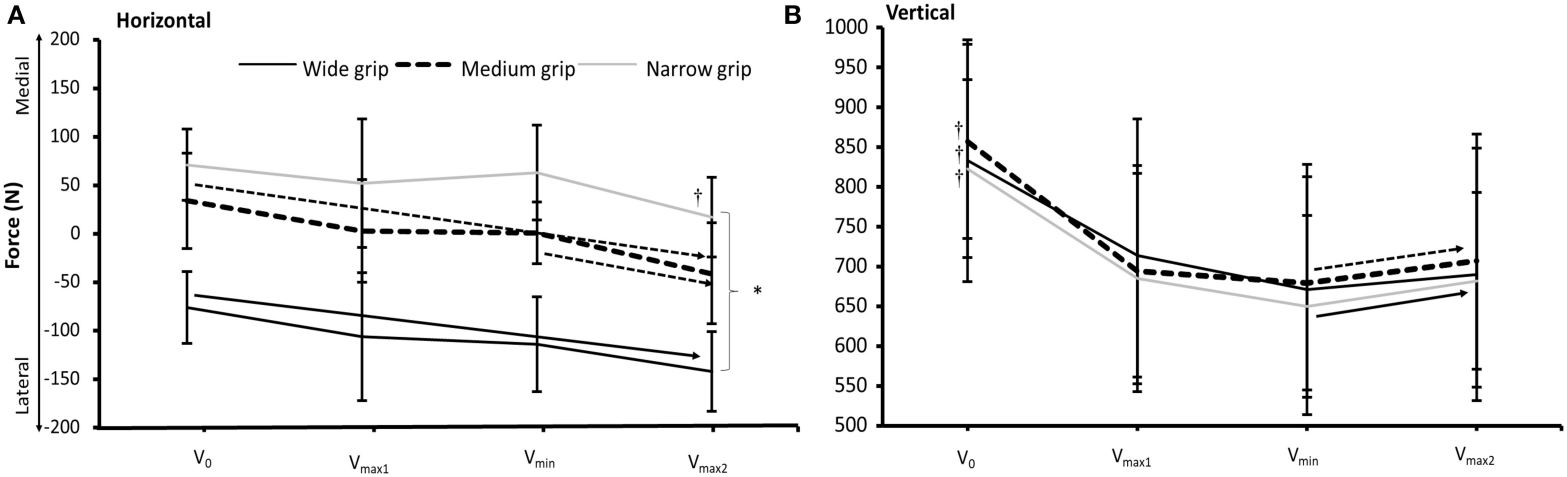
Horizontal forces and vertical forces expressed as mean (SD) in v_0_, v_max1_, v_min_, and v_max2_ for the wide, medium, and narrow grip bench press conditions. *Indicates a significant difference when compared with the other two grip widths. ^†^Indicates a significant difference with all other events for the grip width. → Indicates a significant difference between the two specified events for this grip width.

Significant effects of condition (*F* = 29.2, *p* < 0.001, η^2^ = 0.75) and event (*F* = 19.2, *p* = 0.001, η^2^ = 0.66) were found for horizontal force. *Post hoc* comparison revealed a significant difference in horizontal forces between all three bench press conditions. Furthermore, the horizontal forces acted laterally on the wide bench press condition and medially on the medium and narrow bench press conditions (Figure 4). The lateral forces increased from v_0_ to v_min_ and v_max2_ for the wide bench press condition. For the medium and narrow bench press conditions, medial directed forces decreased from v_0_ to v_max2_ and from v_min_ to v_max2_. For the medium bench press condition, the forces changed from medial in v_min_ to lateral in v_max2_ (Figure 4).

For the vertical forces, only a significant effect of event was found (*F* = 43.4, *p* < 0.001, η^2^ = 0.81). *Post hoc* comparison revealed that vertical forces decreased from v_0_ to all three events that occurred later in the ascent during all bench press conditions, and increased again from v_min_ region to v_max2_ for all three bench press conditions.

**Figure 5:**
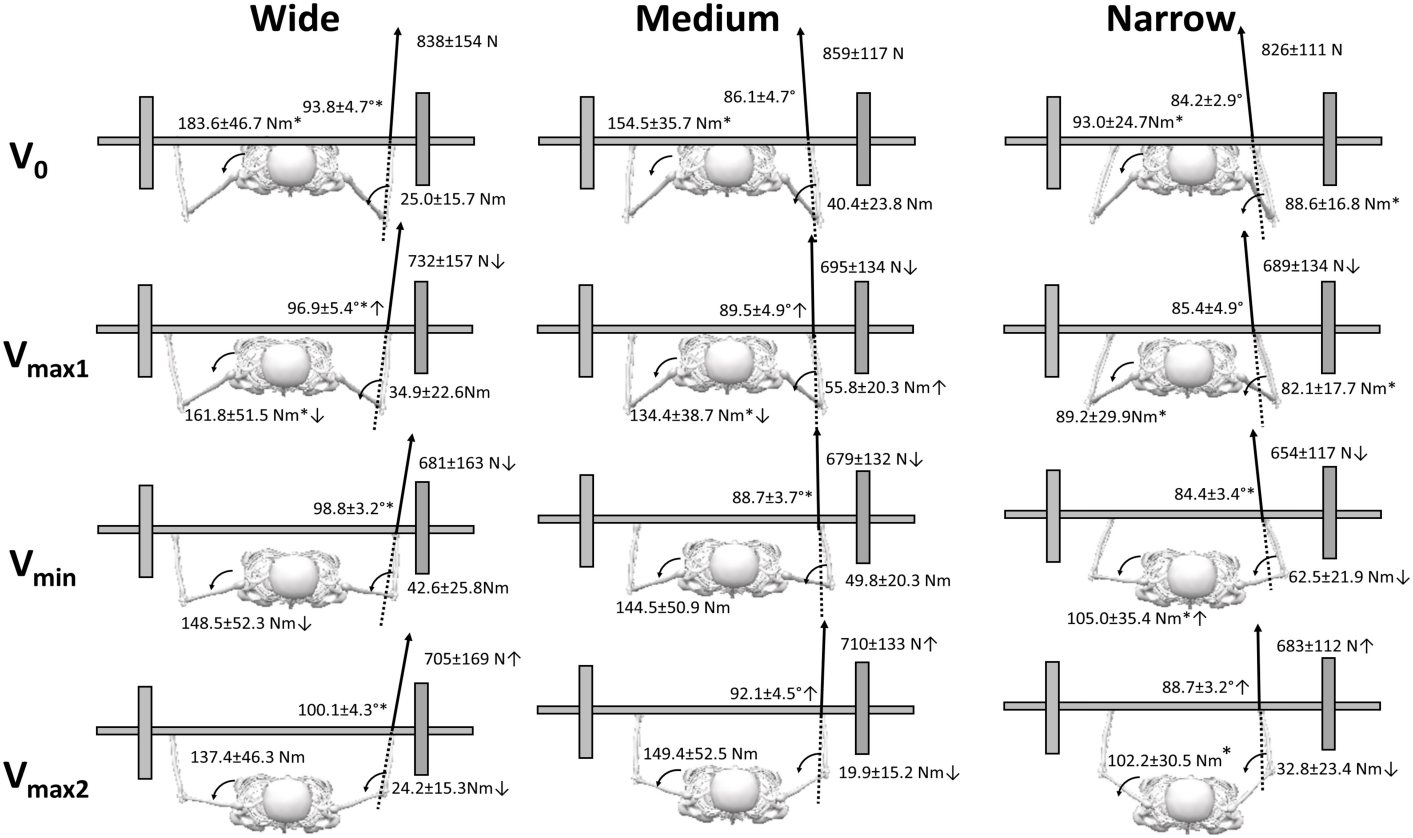
Resultant force, direction of the resultant force vector in the transversal plane, and moment around elbow and shoulder during the events V_0_, v_min_, v_max2_, v_0_, and v_max1_ for the wide, medium, and narrow grip bench press conditions. *Indicates a significant difference when compared with the other two grip widths. ^†^Indicates a significant difference with all other events for this grip width. → Indicates a significant difference between the two specified events for this grip width.

The resultant force was affected by event (*F* = 42.3, *p* < 0.001, η^2^ = 0.81), but not by grip condition (*F* = 0.4 *p* = 0.68, η^2^ = 0.04). However, a significant effect of condition (*F* = 38.7, *p* < 0.001, η^2^ = 0.8), event (*F* = 15.8, *p* < 0.001, η^2^= 0.61), and a condition^*^event interaction effect (*F* = 2.7, *p* = 0.023, η^2^ = 0.21) were found for the resultant force direction (angle). *Post hoc* tests revealed that the resultant force was the highest at v_0_ and decreased in each event to v_min_, after which it increased again in all three conditions (Figure 5). The resultant force angle was significantly greater for the wide grip when compared to the medium and narrow grips, which signifies that the force acted laterally on the wide grip, more neutrally on the medium grip, and medially on the wide grip (Figure 5). Furthermore, the resultant force angle increased significantly between events to v_max2_; more specifically, it increased in the wide and medium grips significantly from v_0_ to v_max1_, and for the narrow and medium grips from v_min_ to v_max2_ (Figure 5).

The differences in angle of the resultant force between the three grip conditions at the different events caused significant effects of condition (*F* ≥ 13.1, *p* < 0.001, η^2^ ≥ 0.62), event (*F* = 5.7, *p* ≤ 0.004, η^2^ ≥ 0.42), and interaction condition^*^event (*F* ≥ 6.2, *p* < 0.001, η^2^ ≥ 0.44) on both moment arms around the elbow and shoulder (Table 2). *Post hoc* comparison revealed that the moment arms on the shoulder were significantly larger from wide to medium to small grip at v_0_ and v_min_, while the opposite was observed on the elbow, with a larger moment arm from small to medium to wide grip. The moment arm around the elbow increased from v_0_ to v_max1_ and decreased again from v_min_ to v_max2_, whereas the moment arm on the shoulder was stable for the first two events and increased for the medium and narrow grips at v_min_, but decreased in the wide and narrow grips from v_min_ to v_max2_ (Table 2). This resulted in significant effects of condition (*F* ≥ 14.6, *p* < 0.001, η^2^ ≥ 0.71), event (*F* ≥ 4.5, *p* ≤ 0.013, η^2^ ≥ 0.36), and condition^*^event interaction (*F* ≥ 7.6, *p* ≤ 0.003, η^2^ ≥ 0.56) on both shoulder and elbow joints. *Post hoc* comparison showed that in all conditions, elbow flexion and shoulder extension occurred during the lifts. The flexion moment on the elbow was significantly higher for the narrow grip during v_0_ and v_max1_ events as compared to the other grip conditions. The shoulder extension moment was larger with each increasing grip width at the first two events, and the shoulder extension was still significantly lower for the narrow condition at v_min_ and v_max1_ as compared with the other two conditions. The elbow flexion moment changed in different ways between the conditions: with the medium grip, the flexion moment increased first from v_0_ to v_max1_ and decreased again from v_min_ to v_max2_, but it decreased in each event from v_min_ in the narrow grip. The extension moment on the shoulder decreased from v_0_ to v_max1_ in the wide and medium grip conditions and continued to decrease to the next event for the wide condition, whereas it increased from v_max1_ to v_min_ in the narrow grip condition (Figure 5).

**Table 2:** Mean (±SD) moment arms on elbow and shoulder at the different events in the wide, medium, and narrow grip bench press.

**Event**	**Grip condition**	**Moment arm elbow (m)**	**Moment arm shoulder (m)**
v_0_	Wide	0.00 ± 0.02^*^	0.24 ± 0.03^*^
	Medium	0.03 ± 0.02^*^	0.19 ± 0.03^*^
	Narrow	0.08 ± 0.03^*^	0.12 ± 0.03^*^
v_max1_	Wide	0.04 ± 0.03^↑^	0.23 ± 0.04^*^
	Medium	0.06 ± 0.02^↑^	0.18 ± 0.04^*^
	Narrow	0.09 ± 0.04^*↑^	0.13 ± 0.03^*^
v_min_	Wide	0.06 ± 0.02	0.22 ± 0.03
	Medium	0.06 ± 0.02	0.22 ± 0.03^↑^
	Narrow	0.07 ± 0.02	0.17 ± 0.05^*↑^
v_max2_	Wide	0.01 ± 0.01^↓^	0.19 ± 0.02^↓^
	Medium	0.01 ± 0.02^↓^	0.19 ± 0.03^↓^
	Narrow	0.02 ± 0.02^↓^	0.15 ± 0.06

* *Indicates a significant difference with all other grip conditions on a p < 0.05 level*.

*Indicates a significant difference between these two grip conditions on a p < 0.05 level*.

*^↓^ or ^↑^ indicates a significant increase or decrease with the pervious event for this condition on a p < 0.05 level*.

### Electromyography

A significant effect of region was found for the deltoideus posterior (*F* = 14.6, p < 0.001, η^2^ = 0.59), the medial triceps (F = 12.84, p < 0.001, η^2^ = 0.59), and lateral triceps (F = 5.36, p = 0.033, η^2^ = 0.35). *Post hoc* tests demonstrated an increase in deltoideus posterior muscle activity through the regions (Table 3). Furthermore, the medial triceps increased muscle activity through the regions for the wide and medium bench press conditions, while the lateral triceps increased muscle activity from the pre-sticking to the post-sticking region. No significant effect of condition was found for any of the muscles except for the medial triceps, for which the *post hoc* test revealed greater muscle activity for the medium and narrow grips than the wide grip bench press condition.

**Table 3:** Mean RMS (SD) electromyography activity of eight upper limb muscles in the pre-sticking, sticking and post-sticking region of the wide, medium, and narrow grip bench press.

**Muscle (μV)**	**Condition**	**Regions**			**Sign between regions**	**Sign between conditions**
		**Pre-sticking**	**Sticking**	**Post-sticking**		
Pectoralis major upper	Wide grip	733 ± 483	728 ± 514	752 ± 479		
	Medium grip	786 ± 598	718 ± 529	614 ± 333		
	Narrow grip	751 ± 596	700 ± 583	706 ± 479		
Pectoralis major lower	Wide grip	514 ± 484	623 ± 785	679 ± 730		
	Medium grip	602 ± 523	598 ± 532	575 ± 454		
	Narrow grip	581 ± 492	639 ± 648	562 ± 487		
Triceps medialis	Wide grip	571 ± 247	871 ± 301	978 ± 417	Pre-sticking with all others	With medium and narrow
	Medium grip	689 ± 365	1006 ± 306	1137 ± 507	Pre-with post-sticking	
	Narrow grip	812 ± 281	847 ± 305	1040 ± 420		
Triceps lateralis	Wide grip	472 ± 436	602 ± 477	702 ± 459	Pre- with post-sticking	
	Medium grip	494 ± 337	593 ± 283	716 ± 450		
	Narrow grip	518 ± 337	611 ± 443	657 ± 562		
Deltoideus anterior	Wide grip	893 ± 573	921 ± 615	1089 ± 801		
	Medium grip	808 ± 485	925 ± 615	1004 ± 694		
	Narrow grip	772 ± 484	874 ± 585	950 ± 721		
Deltoideus medius	Wide grip	275 ± 388	297 ± 420	377 ± 547		
	Medium grip	238 ± 308	278 ± 298	274 ± 332		
	Narrow grip	256 ± 252	378 ± 397	356 ± 341		
Deltoideus posterior	Wide grip	161 ± 121	218 ± 123	286 ± 189	Pre-with sticking	
	Medium grip	161 ± 72	213 ± 123	263 ± 129	Pre-with post-sticking	
	Narrow grip	126 ± 47	186 ± 101	234 ± 73	Pre-sticking with all others	
Biceps brachii	Wide grip	609 ± 560	218 ± 258	194 ± 241		
	Medium grip	415 ± 379	117 ± 116	127 ± 74		
	Narrow grip	341 ± 315	163 ± 118	120 ± 64		

## Discussion

The aim of the study was to investigate the effect of grip width on the joint angles, vertical and horizontal forces, resultant force angle and magnitude, joint moment arms, and moments, in tandem with the muscle activation around the sticking region in the 1-RM barbell bench press among recreationally trained males. Despite the participants lifting different 1-RM loads, the total resultant force did not change between the three grip width conditions. Grip width resulted in different shoulder abduction angles in most events, while elbow extension at the start of the lift and shoulder flexion at the later events were different between the three conditions. Furthermore, the horizontal forces were laterally directed in the wide grip, but were directed medially in the medium and narrow grips and consisted of a maximum of 19% of the total force. The changes in direction of resultant force production during the lifts resulted in differences in moments around the elbow and shoulder, more so with the narrow condition than the two other grip widths. Between the grip width conditions, only triceps medialis muscle activity was greater for the medium and narrow grip widths than the wide grip width.

The findings of the present study on barbell and joint kinematics are in accordance with a previous study on grip widths by Gomo and van den Tillaar (2016); however, the resultant moment arms around the shoulder were much lower than in previous studies (Elliott et al., 1989; Gomo and van den Tillaar, 2016), and the resultant moment arms around the elbow were different (Gomo and van den Tillaar, 2016). In the present study, the moment arm around the shoulder increased for the medium and narrow grips in the sticking region and decreased in the following region for the wide and medium grips (Table 2), whereas Gomo and van den Tillaar (2016) showed a decrease in moment arms in these regions for all grips and Elliott et al. (1989) for the wide grip. An explanation for these differences is that the horizontal forces were also used to calculate the resultant force and direction in the present study; as a result, the resultant force decreased the moment arm around the shoulder and changed it around the elbow joint. More specifically, the moment arm increased around the elbow from v_0_ to v_max1_ and decreased again from v_min_ to v_max2_ for all grip widths, whereas Gomo and van den Tillaar (2016) found different developments between the three grip widths and (Elliott et al., 1989) did not report any changes with the wide grip in elbow moment arm during the lift.

These differences in moment arms resulted in greater shoulder extension moments with the wide and medium grip widths than the narrow grip width during all events, while the narrow grip width produced a greater elbow flexion moment during the pre-sticking region (Figure 5). It can therefore be stated that shoulder flexion and elbow extension are used to counteract these moments. It is also speculated that the wide and medium grip widths enable more load to be lifted because they produce greater shoulder flexion moments during all events. A greater shoulder flexion moment could create higher demands on the proximal prime movers, while the narrow grip width could create higher demands on the distal prime movers; however, the only difference in muscle activity between the three grip widths was found in the medial triceps brachii, which was lower in the wide grip condition as compared with the other two.

Furthermore, different developments occurred in both shoulder and elbow moments between the wide and medium grip widths, as compared with the narrow grip width. Where the medium and wide grip widths decreased the shoulder extension moments, the narrow grip width increased the shoulder extension moment during the sticking region. This is because 10.1% of the forces produced acted medially against the barbell for the narrow grip width, creating a lateral resultant force angle of 84.4°, thereby increasing the shoulder extension moment arm and moment at v_min_. The opposite development in shoulder extension moments and moment arms occurred for the wide and medium grip widths. Interestingly, the horizontal force created against the barbell in v_min_ was 15.7% for the wide grip width in the lateral direction and only 1.8% for the medium grip width in the medial direction. This created a resultant force angle of 98.8° and 88.7° for the wide and medium grip widths respectively, which created similar shoulder and elbow moments and moment arm at v_min_. Therefore, since similar moments and moment arms were produced for the wide and medium grip widths at v_min_ despite different angles and grip widths, the occurrence of a sticking region for the wide and medium grip widths may be specific to the joint moments produced rather than specific to angles, as speculated by Gomo and van den Tillaar (2016).

A similar development in resultant force magnitude occurred between the grip widths: the resultant force magnitude decreased from pre-sticking to sticking region before increasing in the post-sticking region. These results are similar to those reported by Elliott et al. (1989) at a 1-RM in bench pressing with a wide grip. However, the current finding suggests that a similar resultant force profile arises during ascent, independent of grip width. Furthermore, the horizontal forces did not increase in the sticking region. Therefore, our hypothesis, which was that the horizontal force output increased in the sticking region while the total force remained the same, did not concur with our findings.

As previously mentioned, this study demonstrated that the wide grip pushed laterally against the barbell while the medium and narrow grip widths pushed medially against the barbell. This created a medial directed resultant force for the wide grip width and a lateral resultant force for the medium and narrow grip widths. Duffey and Challis (2011) found that horizontal forces during maximal bench press lifts were typically around 25% of the vertical force with small fluctuations of <5%. In comparison, the present study found that the horizontal forces varied from 0.4% at v_max1_ with medium grip to 19.5% at v_max2_ with wide grip of the vertical force during bench press, which is a lower ratio than Duffey and Challis (2011) reported. The reason for this discrepancy could be that Duffey and Challis (2011) used novice lifters, whereas the participants in the present study were experienced lifters in the bench press exercise, meaning that they may have lifted with a more effective vertical lifting technique.

Although forces acted in different directions for the grip widths, all events demonstrated an elbow flexor moment during ascent rather than an elbow extension moment, which Gomo and van den Tillaar (2016) speculated could occur when accounting for horizontal forces in the bench press. The fact that the wide and medium grip widths increased lateral forces in the post-sticking region could explain why the triceps activity increased during ascent for these two grip widths while remaining more stable for the narrow grip width (Table 3). A similar development in triceps activity was reported by Lehman (2005), who found an increase in triceps activity when moving from wide to narrower grip widths. Lehman (2005) also found that the level of supination did not influence the increase in triceps brachii muscle activity when moving to a narrower grip width. Hence, based on the kinematic findings from this study, a greater triceps brachii muscle activity could have occurred because the participants in the present study adducted their shoulders (Figure 3) to a greater extent when benching with narrow grip width, which could increase the elbow extension demands to overcome the elbow flexion moment from the barbell. Greater adduction in the shoulders makes the elbow extension demands greater, which is also indicated by a significantly lower elbow extension at the start of the sticking region along with greater triceps activity for the narrow and medium grips. Another finding was that the posterior deltoid muscle activity increased from the pre-sticking to the post-sticking region for all grip widths, which is due to the scapula moving laterally and upwards to push the barbell up during the post sticking region, and because the posterior deltoid is an antagonist muscle that is necessary to stop the ascending movement in a bench press (Sale, 1988).

No significant differences were found between grip widths for the other prime movers' pectoralis major and anterior deltoid. The findings regarding pectoralis muscle activity are partly similar to earlier findings (Barnett et al., 1995; Lehman, 2005; Saeterbakken et al., 2017); one primary difference, however, was that the study by Barnett et al. (1995) found greater muscle activity in the upper pectoralis muscle for the narrow grip width when compared to the wide grip width. This discrepancy in findings may be because Barnett et al. (1995) only investigated the concentric phase and also used similar absolute loads. Additionally, Saeterbakken et al. (2017) found no difference in deltoideus anterior muscle activity between grip widths, which coincides with our findings. Therefore, it is speculated that different grip widths may influence the muscle activity of the distal prime movers to a greater extent than the proximal prime movers.

In this study, only the horizontal forces (lateral/medial) on the barbell and vertical forces were possible to measure in the present set up; furthermore, no force measurements of the horizontal movement of the barbell during the ascend phase could be taken (Madsen and McLaughlin, 1984; Elliott et al., 1989; van den Tillaar and Ettema, 2009, 2010). This is a limitation of the study since the absence of these forces makes it difficult to perform a full three-dimensional inverse dynamics analysis in the bench press.

## Conclusion

When bench pressing with 1-RM loads, wide and medium grip widths allow for more load to be lifted than the narrow grip width among recreationally trained males. Furthermore, the wide grip width creates medially directed resultant forces, the medium grip width creates mainly vertical resultant forces, and the narrow grip width creates laterally directed resultant forces, as well as elbow flexion moments. During the sticking region, all grip widths demonstrated similar horizontal forces, but resultant forces decreased, which suggests that this is a poor biomechanical region despite the grip width that is used in the bench press exercise. The shoulder and elbow moments and moment arms were similar during the sticking region for the wide and medium grip bench press, which makes the occurrence of sticking region for these grip widths specific to the elbow and shoulder joint moments created. When the goal is to lift as much as possible during 1-RM bench press attempts among recreationally trained males, our findings suggest that bench pressing with a wide or medium grip width may be beneficial.

## Data Availability Statement

The raw data supporting the conclusions of this article will be made available by the authors, without undue reservation.

## Ethics Statement

The studies involving human participants were reviewed and approved by Norwegian Center for Research Data project number 991974. The patients/participants provided their written informed consent to participate in this study.

## Author Contributions

RT and OG did the data collection and part of the data analyses, SL did part of the data analysis and writing the first draft, while RT and OG reviewed the manuscript and RT did the supervision over the whole study. All authors contributed to the article and approved the submitted version.

## Conflict of Interest

The authors declare that the research was conducted in the absence of any commercial or financial relationships that could be construed as a potential conflict of interest.
